# Prevalence, perception of risk and mycobacterium tuberculosis control practices among healthcare workers in HIV care and treatment centres in North Central Nigeria

**DOI:** 10.1186/s12879-025-10591-5

**Published:** 2025-02-14

**Authors:** Evaezi Okpokoro, Temitayo Lawal, Oyewole Oyedele, Victoria Etuk, Stella Ijioma, Temitope Adetiba, Miriam Bathnna, Chinye Osa-Afiana, Nifarta Andrew, Alash’le Abimiku

**Affiliations:** 1 International Research Centre of Excellence, Institute of Human Virology Nigeria, Abuja, Nigeria; 2 Division of Epidemiology and Biostatistics, School of Public Health, University of Cape Town, Cape Town, South Africa; 3 Prevention Care and Treatment Department, Institute of Human Virology Nigeria, Abuja, Nigeria; 4 School of Medicine, University of Maryland, Baltimore, MD USA

**Keywords:** Tuberculosis, HIV, Healthcare workers, Risk perception, Nigeria

## Abstract

**Background:**

Globally, Healthcare workers (HWs) are known to be at higher risk of Tuberculosis (TB), especially those working in HIV settings. Understanding HW’s alertness of TB risk is an important step to ensure safety in such settings. Hence, we assessed the perception of the risk of TB and its associated factors among HWs in HIV care and treatment centres in North-Central Nigeria.

**Methods:**

This study is a nested cross-sectional descriptive study conducted among HWs in North-Central Nigeria. HWs were recruited across 14 health facilities with dedicated HIV clinics (*n* = 337). Perception of risk to TB was captured using a binary outcome and a sliding scale of 1–10 on a structured questionnaire, and blood samples were collected and tested for mycobacterium TB infection at the biorepository of the Institute of Human Virology Nigeria. Univariate and Multivariate analysis were performed using STATA version 18, with statistical significance set at 5%.

**Results:**

Overall, 86.9% of HWs in HIV centres perceived themselves to be at risk of TB. Perception of risk to TB was significantly lower among male HWs (crude Odds Ratio: 0.50; 95% Confidence Interval (CI): 0.26–0.95). Findings from the multivariable analysis further revealed that perception of risk to TB was significantly lower among HWs < 20years (adjusted Odds Ratio (aOR): 0.13; 95%CI: 0.02–0.83), HW with no formal/primary education (aOR: 0.10; 95%CI: 0.01–0.73) and HWs working in primary (aOR: 0.24; 95%CI: 0.06–0.92) or secondary (aOR: 0.41; 95%CI: 0.18–0.95) healthcare facilities. Also, daily monitoring of TB Infection Control (IC) practices (aOR: 2.53; 95%CI: 1.27–5.04) and having a designated area for sputum samples collection (aOR: 3.68; 95%CI: 1.38–9.77) were associated with increased odds of perception of risk to TB while the presence of annual HIV testing was associated with decreased perception of risk to TB.

**Conclusion:**

There is a high perception of risk to TB among HWs working in HIV clinics. However, perception of risk to TB is influenced by age, educational status, level of care at healthcare facilities, and level of TB IC practices. TB IC trainings should target younger HWs, who are males, with no formal education and working in primary health facilities.

**Supplementary Information:**

The online version contains supplementary material available at 10.1186/s12879-025-10591-5.

## Introduction

Tuberculosis, (TB) is a bacterial communicable disease that mainly affects the lungs and is the most common opportunistic infection among people living with HIV (PLHIV) [1]. Before the coronavirus pandemic, TB was the leading cause of mortality from a single infectious agent [1, 2]. In 2022, the World Health Organization (WHO) reported that a total of 1.3 million people died from TB (including 167 000 people with HIV)– this implies that 167,000/1,300,000 (12.8%) of deaths among PLHIV are attributable to TB [3]. The global TB report presented a slightly higher estimate of 1.6 million deaths (11.8%) among PLHIV [4], these high number of deaths reflects the significant impact of TB on individuals with HIV/AIDS.

Given, the high percentage of undiagnosed and untreated TB cases among PLHIV [4], there is potentially an increased risk of TB among Healthcare Workers (HWs) or caregivers of PLHIV. More so, previous research has consistently underscored the health risks posed to HWs by occupational exposure to TB [5–7]. In addition, a previous meta-analysis reported that HWs face a threefold greater likelihood of contracting TB compared to the general population [7–9]. Based on the above, we presume that HWs working in HIV clinics are at a heightened risk of occupationally-acquired TB infections in healthcare settings [1, 2].

Given the proximity of HWs to TB infected patients (especially in HIV settings or other high-risk settings), HWs face an elevated risk of contracting this disease especially when proper preventive measures are not implemented [8, 10]. Thus, a significant number of TB cases among HWs can be attributed to exposure within healthcare settings [7–9]. Curtailing this exposure to TB is challenging as most healthcare facilities in low- and middle-income countries do not meet the minimum standards for reducing the risk of occupational acquired infectious diseases– which invariably places HWs at a high risk of being infected [6, 11–13].

Despite the established occupational risk of contracting TB among HWs working in HIV settings [11], it remains unclear from literature how HWs perceive their susceptibility to TB infection and the drivers of their perception. While previous studies have examined the risk perception of HWs working in HIV departments/clinics, these studies did not model the perception of TB risk viz-a-viz their Mycobacterium TB Infection (MTBI) results [14]. We hypothesize that HWs may have poor perception of their risk to TB despite exposure from working in HIV clinics. Here, we report the perception of risk to TB among HWs and correlate this with Interferon gamma release assay (IGRA) test result for TB infection test. In addition, we triangulate TB Infection Control (IC) practice within these HIV clinics to understand how it drives HWs TB risk perception.

## Methods

### Study design and setting

This study is a nested cross-sectional descriptive study conducted among HWs in Healthcare Facilities (HF) with dedicated HIV clinics being funded under the US President’s Emergency Plan for AIDS Relief-funded (PEPFAR) in Federal Capital Territory (FCT) and Nasarawa State. This study is nested within a larger study titled - “Mycobacterium Tuberculosis Infection Rate among HWs in an HIV Care and Treatment Centre in Nigeria (TRACING) Study” funded by the European Developing Countries Clinical Trial Partnership (EDCTP).

### Sample size and data collection

Participant-level data were captured from consenting HWs (clinical and non-clinical) using a standardized and piloted questionnaire (Supplementary File 1). However, prior administrative clearance from the HF as well as HW consent were obtained. The HFs used were purposively selected based on the HIV/TB patient’s caseload. A non-probabilistic, consecutive, sampling approach was employed to recruit consented HWs to participate in the study. Nonetheless, eligibility criteria of HWs included being 18 years and above and being a HW according to the WHO criteria) [15]. Thereafter, a standardized TB risk assessment questionnaire was used to capture the HWs’ demographic characteristics and risk while a modified WHO TB IC assessment form comprising of four components namely: managerial, administrative, environmental, and personal protective equipment was administered. A total of 1043 HWs were recruited into the parent study, but the sample size in this study was 337 (after HWs in non-HIV settings (*n* = 608), and those with missing information/results (*n* = 98) were dropped).

Blood samples were collected in two 5-mL lithium heparin tubes and tested at the biorepository of the Institute of Human Virology Nigeria using the QFT test. A consistent and standardized protocol was adopted by the central biorepository for all processing and testing procedures to minimize batch-to-batch variability. The primary assessment of Mycobacterium TB infection relied on the manufacturer’s suggested threshold of 0.35 IU/mL, which was deemed indicative of a positive test result.

### Statistical analysis

Data were entered into the REDCAP database and exported into STATA version 18 for analysis. Perception of HWs on their TB risk was obtained using the question “*Do you think you are at risk of developing active TB infection in your current work area?*”, with three response options - “Yes” indicating that HWs perceived they were at risk, “No” indicating that HWs perceived that they were not at risk of contracting TB, and “don’t know” indicating that HWs were unsure of their risk. Univariable (frequencies and proportions) and Bivariable (Fisher’s exact [16]) analysis were carried out. A multivariable analysis of all variables was done using Binary Logistics Regression Analysis, with only two outcomes of the dependent variable (Yes and No). Prior to the multivariable analysis, a test of multicollinearity was performed using the “estat vif” command in STATA, and variables with Variance Inflation Factor (VIF) ≥ 5 were dropped from the multivariable analysis. Statistical significance was established at 5%.

## Results

### Baseline characteristics and association with perception of risk to TB

A total of 337 HWs were enrolled for this study from 13 of the 14 HIV care and treatment HFs selected. Majority of the HWs were female (58.2%) and aged 30–39 years (44.9%) (Table 1). Over two-thirds of the HWs were married (71.8%) and over three-quarters had tertiary education (79.5%). Of all the HWs, 49.3% practiced in tertiary health facilities, and 45.1% in secondary health facilities.

Overall, 86.9% of HW perceived being at risk of contracting TB. Data disaggregation showed that the perception of being at risk of contracting TB was significantly lower among male HWs (82.3%) compared to female HWS (90.3%). Also, TB risk perception was lowest among HWs aged < 20 years (55.6%) and HWs with no formal/primary education (70.0%). In addition, health facilities offering primary services (79.0%) had lower risk perception compared to secondary (86.4%) and tertiary education (87.7%). Also, perception of risk to TB was lower among non-clinical HWs (86.5%) compared to clinical HWs (89.3%), although these findings were not statistically significant. The overall prevalence of MTBI using IGRA positive result among HWs in HIV clinics was 42.8%.

**Table 1:** Socio-demographic characteristics of study participants (HWs)

	Overall *n* (col %)	Perception of risk to TB		$\:{\chi\:}^{2}$ (*p*-value)
		Not at risk *n* (row %)	At risk *n* (row %)	
**Age group**				
< 20 years	9 (2.7)	4 (44.4)	5 (55.6)	8.041 (0.090)
20–29 years	53 (15.8)	6 (11.3)	47 (88.7)	
30–39 years	151 (44.9)	19 (12.6)	132 (87.4)	
40–49 years	82 (24.4)	10 (12.2)	72 (87.8)	
50 years and above	41 (12.2)	5 (12.2)	36 (87.8)	
**Gender**				
Female	196 (58.2)	19 (9.7)	177 (90.3)	**4.666 (0.031)**
Male	141 (41.8)	25 (17.7)	116 (82.3)	
**Educational Qualification**				
No formal education/Primary	10 (3.0)	3 (30.0)	7 (70.0)	2.673 (0.263)
Secondary education	59 (17.5)	8 (13.6)	51 (86.4)	
Tertiary education	268 (79.5)	33 (12.3)	235 (87.7)	
**Marital Status**				
Single/Previously married	95 (28.2)	11 (11.6)	84 (88.4)	0.279 (0.870)
Married	242 (71.8)	33 (13.6)	209 (86.4)	
**Facility level of care**				
Primary	19 (5.6)	4 (21.0)	15 (79.0)	2.806 (0.246)
Secondary	152 (45.1)	23 (15.1)	129 (84.9)	
Tertiary	166 (49.3)	17 (10.2)	149 (89.8)	
**Employment Designation**				
Non-Clinical	56 (16.6)	6 (10.7)	50 (89.3)	0.325 (0.569)
Clinical	281 (83.4)	38 (13.5)	243 (86.5)	
**QFT-Result** (***N*** = **285**^**e**^)				
Positive	122 (42.8)	19 (15.6)	103 (84.4)	0.645 (0.422)
Negative	163 (57.2)	20 (12.3)	143 (87.7)	

QFT– QuantiFERON plus; ^e^– 52 indeterminate results excluded; bold *p*-values implies significance at 5%

**Fig. 1:**
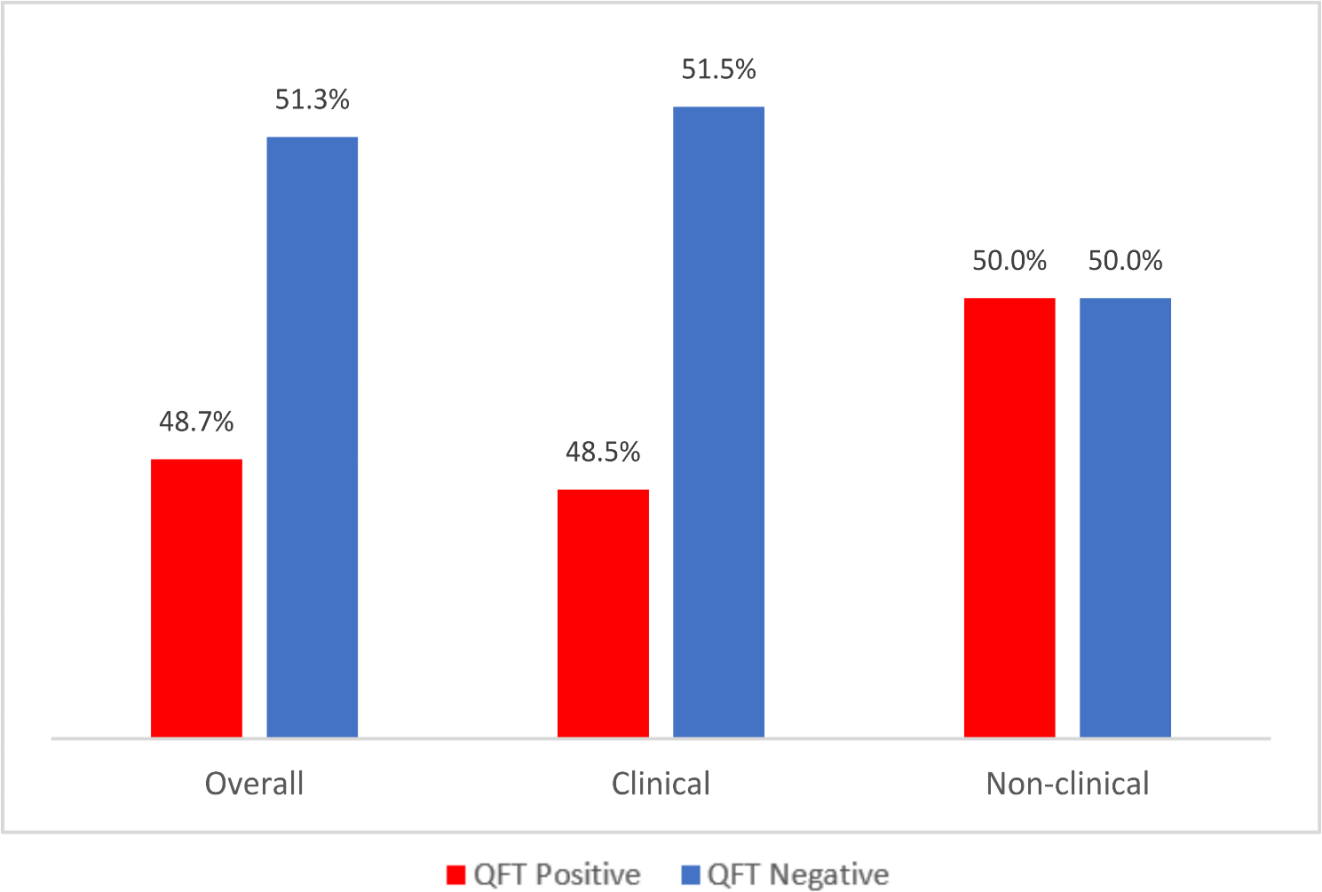
QuantiFERON result among HWs with no perceived risk to TB

**Fig. 2:**
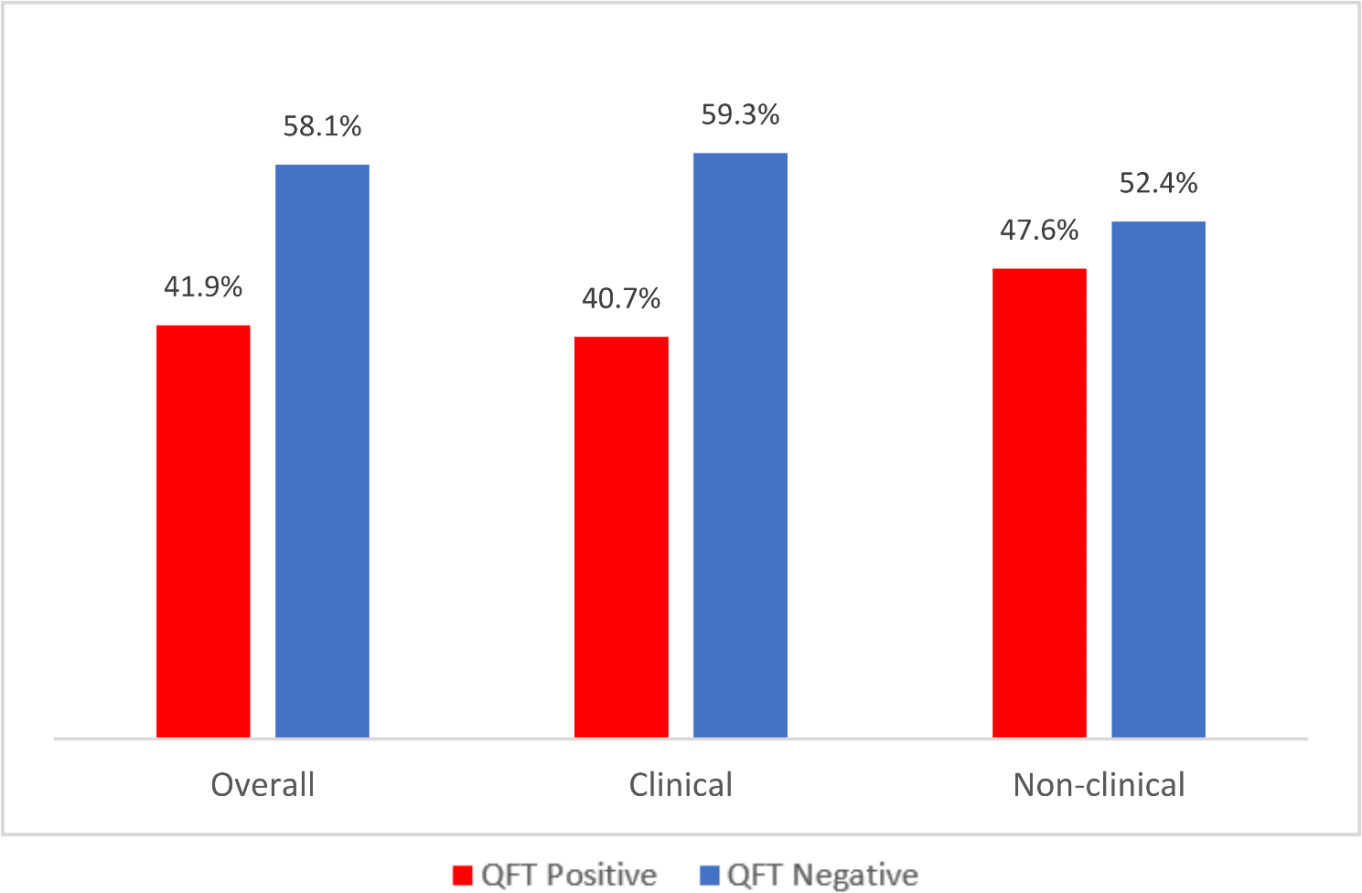
QuantiFERON result among HWs with perceived risk to TB

As shown in the Figure, there is a higher prevalence of TB Infection using IGRA positive results among HWs in HIV clinics who reported not being at risk of TB (Fig. 1) compared to those reportedly at risk of TB (Fig. 2) (i.e. 48.7% vs. 41.9%). When stratified by employment designation, (non-clinical vs. clinical HW), across both groups (“at risk” and “not at risk”), a slightly higher percentage of non-clinical HWs had positive IGRA results compared to the percentage of clinical staff with positive IGRA results (Figs. 1 and 2).

### Association between TB infection control practices and perception of risk to TB

As shown in Table 2, perception of risk to TB was higher among HWs whose HF had the practice of conducting daily monitoring of TB IC practices ($\:{\chi\:}^{2}$ = 4.666; *p*-value = 0.031). Also, perception of risk to TB was higher among HW whose HF did not conduct the following TB IC elements namely: occupational health program ($\:{\chi\:}^{2}$ = 4.443; *p*-value = 0.035) nor offer annual HIV tests to staff ($\:{\chi\:}^{2}$ = 6.513, *p*-value = 0.011).

**Table 2:** TB infection control among HWs stratified by perception of risk to TB

TB Infection Control Activities	Overall *N* (col %)	Risk Perception		$\:{\chi\:}^{2}$ (*p*-value)
		Not at risk *n* (row %)	At risk *n* (row %)	
**MANAGERIAL DOMAIN**				
**Daily monitor TB IC practices**				
No	141 (41.8)	25 (17.7)	116 (82.3)	**4.666 (0.031)**
Yes	196 (58.2)	19 (9.7)	177 (90.3)	
**Has a designated infection control committee**				
No	67 (19.9)	7 (10.4)	60 (89.6)	0.501 (0.479)
Yes	270 (80.1)	37 (13.7)	233 (86.3)	
**Conducts operational research to improve TB IC**				
No	263 (78.0)	31 (11.8)	232 (88.2)	1.700 (0.192)
Yes	74 (22.0)	13 (17.6)	61 (82.4)	
**Facility has an occupational health program**				
No	305 (90.5)	36 (11.8)	269 (88.2)	**4.443 (0.035)**
Yes	32 (9.5)	8 (25.0)	24 (75.0)	
**ADMINISTRATIVE DOMAIN**				
**Coughing patients are separated and fast-tracked**				
No	14 (4.2)	4 (28.6)	10 (71.4)	3.097 (0.078)
Yes	323 (95.9)	40 (12.4)	283 (87.6)	
**Has a designated area for sputum samples collection**				
No	29 (8.6)	7 (24.1)	22 (75.9)	3.433 (0.064)
Yes	308 (91.4)	37 (12.0)	271 (88.0)	
**INH preventive treatment is offered to staff**				
No	49 (14.5)	10 (20.4)	39 (79.6)	2.730 (0.098)
Yes	288 (85.5)	34 (11.8)	254 (88.2)	
**Staff are offered HIV test annually**				
No	304 (90.2)	35 (11.5)	269 (88.5)	**6.513 (0.011)**
Yes	33 (9.8)	9 (27.3)	24 (72.7)	
**ENVIRONMENTAL DOMAIN**				
**Regular maintenance of extractor fan**				
No	297 (88.1)	39 (13.1)	258 (86.9)	0.012 (0.911)
Yes	40 (11.9)	5 (12.5)	35 (87.5)	
**Servicing documentation is maintained**				
No	278 (82.5)	39 (14.0)	239 (86.0)	1.323 (0.250)
Yes	59 (17.5)	5 (8.5)	54 (91.5)	
**Waiting areas have good cross ventilation**				
No	82 (24.3)	12 (14.6)	70 (85.4)	0.238 (0.626)
Yes	255 (75.7)	32 (12.5)	223 (87.5)	
**PPE DOMAIN**				
**Surgical masks are available and worn by patients**				
No	208 (61.7)	28 (13.5)	180 (86.5)	0.079 (0.779)
Yes	129 (38.3)	16 (12.4)	113 (87.6)	
**N95 or FFP respirators are available**				
No	286 (84.9)	37 (12.9)	249 (87.1)	0.024 (0.878)
Yes	51 (15.1)	7 (13.7)	44 (86.3)	
**Trained staff on proper fit of respirators**				
No	263 (78.0)	31 (11.8)	232 (88.2)	1.700 (0.192)
Yes	74 (22.0)	13 (17.6)	61 (82.4)	

PPE– Personal protective equipment; bold *p*-values implies significance at 5%

### Factors associated with the perception of risk to TB among HWs

As shown in Table 3, the perception of the risk of TB among HWs increased with advancing age. Thus, the perception of risk of TB was lower among HWs below 20 years compared to those aged 50 years and above [adjusted Odds Ratio (aOR): 0.13; 95% Confidence Interval (CI): 0.02–0.83]. Likewise, HWs with no formal education/primary education had a lower perception of risk of TB compared to HWs with tertiary education (aOR: 0.10; 95% CI: 0.01–0.73). HWs working in a health facility that provides primary or secondary services were associated with a lower perception of risk to TB compared to those in tertiary health facilities (aOR: 0.24, 0.41; 95% CI: 0.06–0.92, 0.18–0.95 respectively). It is worth noting that the odds of TB risk perception differed by employment designation.

**Table 3:** Multivariable logistics regression of perception of risk to TB among HWs

	Crude Odds Ratio (95% CI)	Adjusted Odds Ratio (95% CI)
**SOCIO-DEMOGRAPHIC CHARACTERISTICS**		
**Age group**		
< 20 years	0.17 (0.03, 0.87) *	0.13 (0.02, 0.83) *
20–29 years	1.09 (0.31, 3.85)	0.69 (0.14, 3.30)
30–39 years	0.96 (0.34, 2.76)	0.78 (0.23, 2.69)
40–49 years	1.00 (0.32, 3.14)	0.89 (0.23, 3.40)
50 years and above	Reference	
**Gender**		
Female	Reference	
Male	0.50 (0.26, 0.95) *	0.62 (0.30, 1.28)
**Educational Qualification**		
No formal education/Primary	0.33 (0.08, 1.33)	0.10 (0.01, 0.73) *
Secondary education	0.90 (0.39, 2.05)	0.55 (0.20, 1.46)
Tertiary education	Reference	
**Marital Status**		
Single/Previously married	Reference	
Married	0.83 (0.40, 1.72)	0.79 (0.31, 2.04)
**Facility level of care**		
Primary	0.43 (0.13, 1.44)	0.24 (0.06, 0.92) *
Secondary	0.64 (0.33, 1.25)	0.41 (0.18, 0.95) *
Tertiary	Reference	
**Employment Designation**		
Non-Clinical	Reference	
Clinical	0.77 (0.31, 1.91)	0.50 (0.13, 1.93)
**QFT-Result**		
Negative	Reference	
Positive	0.76 (0.39, 1.49)	0.88 (0.42, 1.81)
**MANAGARIAL DOMAIN**		
**Daily monitor TB IC practices**		
No	Reference	
Yes	2.01 (1.06, 3.81) *	2.53 (1.27, 5.04) *
**Has a designated infection control committee**		
No	Reference	
Yes	0.73 (0.31, 1.73)	0.55 (0.21, 1.40)
**Conducts operational research to improve TB IC**		
No	Reference	
Yes	0.63 (0.31, 1.27)	0.68 (0.32, 1.43)
**Facility has an occupational health program**		
No	Reference	
Yes	0.40 (0.17, 0.96)	0.51 (0.20, 1.27)
**ADMINISTRATIVE DOMAIN**		
**Coughing patients are separated and fast-tracked**		
No	Reference	
Yes	2.83 (0.85, 9.45)	1.93 (0.45, 8.28)
**Has a designated area for sputum samples collection**		
No	Reference	
Yes	2.33 (0.93, 5.83)	3.68 (1.38, 9.77) *
**INH preventive treatment is offered to staff**		
No	Reference	
Yes	1.92 (0.88, 4.18)	2.39 (0.88, 6.51)
**Staff are offered HIV test annually**		
No	Reference	
Yes	0.35 (0.15, 0.81) *	0.23 (0.09, 0.57) *
**ENVIRONMENTAL DOMAIN**		
**Regular maintenance of extractor fan**		
No	Reference	
Yes	1.06 (0.39, 2.86)	1.51 (0.51, 4.53)
**Servicing documentation is maintained**		
No	Reference	
Yes	1.76 (0.66, 4.68)	2.42 (0.80, 7.26)
**Waiting areas have good cross ventilation**		
No	Reference	
Yes	1.19 (0.58, 2.44)	1.72 (0.74, 4.00)
**PPE DOMAIN**		
**Surgical masks are available and worn by patients**		
No	Reference	
Yes	1.10 (0.57, 2.12)	1.10 (0.56, 2.12)
**N95 or FFP2 respirators are available**		
No	Reference	
Yes	0.93 (0.39, 2.23)	2.23 (0.66, 7.62)
**Trained staff on proper fit of respirators**		
No	Reference	
Yes	0.63 (0.31, 1.27)	0.38 (0.14, 1.03)

TB IC– Tuberculosis Infection Control; HIV– Human Immunodeficiency Virus; INH– Isoniazid; PPE– Personal Protective Equipment; N95– Non-Oil 95 mask; FFP– filtering facepiece; * - significant at 5%

Furthermore, findings from the result show that HWs in facilities where TB IC practices are monitored daily (aOR: 2.53; 95% CI: 1.27–5.04) and facilities that have a designated area for sputum samples collection (aOR: 3.68; 95% CI: 1.38–9.77) had higher perception of risk to TB. The staff of health facilities that offer annual HIV testing had a lower perception of TB risk infection compared to HWs whose facilities offer no HIV testing (aOR: 0.23; 95% CI: 0.09–0.57).

## Discussion

HWs are often at an increased risk of developing infections due to their proximity to infected patients, and this risk is usually higher in high-risk settings [2, 9]. To this end, our study measured the perception of risk towards acquisition of TB and correlates this with the actual TB infection rates among HW working in HIV clinics as well as the association of their perception of risk and TB IC practices within the HF. We found that a high proportion of these HWs considered themselves as being at risk of contracting TB. Notably, more HWs who did not perceive themselves to be at risk tested positive for TB compared to those who did. Several factors such as background TB exposure, literacy levels and sociodemographic factors account for divergent perception of TB among HWs– the observed correlation between TB perception and other factors may be responsible for the varied uptake of TB preventive services despite the huge TB infection rates and the implementation of the WHO guidelines to provide HWs with TB exposure or latent TB infection with preventive therapy [4].

### Perceived risk of TB Infection and MTBI results of HWs in HIV settings

Based on our study, a high proportion of HW (86.9%) working in the HIV clinic consider themselves being at risk of contracting TB. However, only 42.8% of HW with valid results were positive for MTBI based on IGRA testing. In a similar study conducted among HWs in South Africa, van Rie et al., found that 52.9% of HWs perceived their risk to TB as likely or highly likely to have TB [14], while 76.9% reported the same in Swaziland [12]. However, there is no report on the prevalence of TB infection among the respondents in these studies.

Despite the established prevalence and risk of TB among PLHIV, it’s noteworthy that the study found that not all HWs in this setting perceive themselves as being at risk of contracting TB. Notable is that a higher proportion of HWs who had considered themselves not to be at risk of TB were positive for MTBI compared to those who perceived themselves to be at risk (48.7% vs. 41.9%). This apparently inverse correlation between perception of risk and positive result of TB infection may be due to the differing level of knowledge on TB. Although previous literatures have noted that there is no significant difference in the risk of contracting infection among clinical versus nonclinical staff [9, 17], since clinical HWs may be more accustomed to strict infection control practices (including the use of personal protective equipment), it is anticipated that clinicians (doctors, nurses, lab scientist) in comparison with nonclinical staff (i.e., admin, cleaners, security officers etc.) would exhibit higher knowledge and be more alert to their risk to TB.

In our previous publication, we reported a higher prevalence of TB infection among nonclinical staff which may be linked to their lower knowledge on TB as well as other factors such as living in densely populated neighbourhood as a surrogate to socioeconomic status [2]. Thus, it becomes imperative that the TB education and awareness targets nonclinical personnel given that this population may be at a higher risk of TB compared to clinical personnel. In addition, understanding the perception of risk is critical in developing behavioural change intervention towards curtailing the occupationally acquired TB. It is plausible that, since these HWs do not perceive themselves as being at risk of TB, they would not seek TB testing or preventive therapy. This scenario presents a stumbling block to the TB IC efforts, potentially allowing the TB infection to progress to active disease and increasing TB transmission risk within HWs and to other high-risk individuals– such as in an HIV setting [9, 18].

### Factors associated with TB risk perception

Several factors are associated with perception of risk to TB. It is established that education plays a pivotal role in shaping healthcare perception [19]. Previous studies have reported that there is a relationship between poor disease knowledge/perception and low educational levels [19, 20]. Health perception, in turn, affects the health-seeking behaviours and health responsibility of individuals– individuals with higher education levels often have higher health literacy, enabling them to have a better understanding of healthcare outcomes [19, 21]. This study corroborates this finding, and findings from previous studies [12, 22], by demonstrating that HWs in HIV settings with higher levels of education were more likely to perceive themselves as being at risk of contracting TB.

Similar to our finding, previous literature on a cumulative meta-analysis of 12 studies [13] that consisted of 2871 cases and 15,673 controls found that younger HWs (< 30 years) had a significantly lower risk of TB perception (OR: 0.56: 95% CI: 0.40–0.80, *p* = 0.002). It is well known that age plays a very important role in the health risk perception of adults as young individuals may perceive themselves as invulnerable to health risks [13, 23]. This perception can lead to a lack of concern about infectious diseases like TB because younger individuals may believe that such diseases primarily affect older or more vulnerable populations. We also posit that this perception may be entrenched in their lack of experience and educational attainment [5].

Previous studies have established that the implementation of TB IC practices and measures such as monitoring of TB IC practices [9, 24] and staff screening [8, 9] could contribute to about 81% reduction in the incidence of TB among HWs in high-risk settings [9]. Regular monitoring of TB IC practices in health facilities would help in the identification of gaps and/or areas of weaknesses in TB IC practices that require improvement, and this would contribute to an increased awareness of HWs about their risk of contracting TB infection.

Furthermore, monitoring of TB IC practices could also encourage HWs to adhere to established guidelines more diligently than they normally would [6, 25]. In addition to monitoring, the presence of a designated area for sputum collection may continually remind HWs of the importance of infection control measures [1, 6, 12]. This would serve as a constant reminder that they are at risk of contracting TB and should consistently employ and follow proper infection control protocols.

In this study, we further found that having a designated infection control commute, conducting operational research to improve TB IC and having an occupational health program may reduce the perception of HWs to TB risk. We posit that though these components may enhance the health and safety of HWs– however, there may be a possibility that better safety measures can lead to reduced TB risk perception.

### TB IC practices of the facilities

Across facility levels of care, the study found significant differences in TB IC practices. It is expected that healthcare facilities at higher levels would handle more severe cases of TB and visit frequency, which could influence the intensity of TB IC measures [26]. Also, the availability of resources may differ across different levels of healthcare, especially in low-resource settings [24, 26]. However, irrespective of the level of TB services offered, it is important that health facilities ensure the prioritization and implementation of TB IC interventions and occupational health programmes, which include robust monitoring and evaluation. This is critical to reduce TB transmission to patients and HWs. The provision of preventive therapy for HWs with latent TB infection (LTBI) can also prevent progression to active TB.

## Conclusion and recommendation

Many people living with HIV have weakened immune systems due to the virus’ impact on CD4 T-cell counts and are therefore more susceptible to various opportunistic infections like TB. HWs play a crucial role in the elimination of TB and the recognition of their risk is a significant starting point. Our study showed that there is high alertness of the perceived risk to TB among HWs in HIV clinics, but some HWs still do not perceive themselves as being at risk to TB. This gap was associated with their level of education, age, and facility level of care. The presence of certain elements of the TB IC practices were also associated with perception of risk to TB– the presence of daily TB IC monitoring, and designated sputum areas were associated with increased perception of risk while the presence of annual HIV testing were associated with decreased perception of risk to TB.

Furthermore, the high rate of TB infection among HWs in HIV settings highlights a high level of occupational health risk which may be connected to the observed poor implementation of TB IC activities and practices. Given the high level of occupational exposure and the risk of further transmission to patients, prioritization of TB IC interventions, coupled with rigorous surveillance and monitoring systems should be put in place to achieve the national TB elimination plan among both clinical and non-clinical HWs in HIV clinics. Behavioural change models that focus on TB literacy and TB IC practices are strategic interventions that should be embraced towards curtailing the risk to TB infection among HW in HIV clinics.

### Strength and limitation

The strength of this study lies in the exploration of the perception of risk to TB among HWs in HIV settings in order to recommend interventions that could curb the spread of infections. Given that this study is nested, our measure of TB risk perception was limited to the use of only one question to evaluate HW’s perception of risk, which may limit our findings. Also, the nonprobabilistic approach to sampling may limit our findings, however, to minimize this bias, we increased our study recruitment sites to 13 health facilities with the highest HIV/TB patent’s caseload.

## Electronic supplementary material

Below is the link to the electronic supplementary material.

Supplementary File 1: TB risk questionnaire

## Data Availability

The datasets used and/or analysed during the current study are available from the corresponding author on reasonable request.

## Abbreviations

TB: Tuberculosis
HW: Healthcare workers
OR: Odds Ratio
CI: Confidence Interval
PPE: Personal protective equipment
TB IC: Tuberculosis Infection Control
PLHIV: People Living with HIV
MTBI: Mycobacterium tuberculosis
HIV: Human Immunodeficiency Virus
IGRA: Interferon-Gamma Release Assays
QFT: QuantiFERON plus
