# Supplementary material for: Prevalence, perception of risk and mycobacterium tuberculosis control practices among healthcare workers in HIV care and treatment centres in North Central Nigeria
Source: BMC Infect Dis. 2025 Feb 14;25:220. doi: 10.1186/s12879-025-10591-5 (PMC11829344; doi:10.1186/s12879-025-10591-5)
Supplement: Supplementary file 1 — TB risk questionnaire [file 12879_2025_10591_MOESM1_ESM.pdf]

Data Dictionary Codebook

01.04.2023 19:00

#	Variable / Field Name	Field Label<br>*Field Note*	Field Attributes (Field Type, Validation, Choices, Calculations, etc.)
Instrument: **Eligibility Criteria Form** (eligibility_criteria_form)			
1	[participant_id]	Participant ID	text
2	[date_elg]	Date	text (date_dmy)
3	[signed_consent]	Section Header: *INCLUSION CRITERIA*<br>Signed informed Consent	dropdown<br>0 No<br>1 Yes
4	[age_18]	Age (>_18)	dropdown<br>0 No<br>1 Yes
5	[ever_had_tuberculosis]	Section Header: *EXCLUSION CRITERIA*<br>Have you ever had tuberculosis	dropdown<br>0 No<br>1 Yes<br>2 Don't know
6	[have_tuberculosis]	Do you Currently have tuberculosis	dropdown<br>0 No<br>1 Yes<br>2 Don't know
7	[treated_for_tb]	Is anyone living with you being treated for TB	dropdown<br>0 No<br>1 Yes
8	[immunosuppressive_agents]	Are you currently undergoing any therapy with immunosuppressive agents	dropdown<br>0 No<br>1 Yes<br>2 Don't know
9	[days_after_vaccination]	Are you less than 30 days after any vaccination	dropdown<br>0 No<br>1 Yes<br>2 Dont know
10	[latent_tb_infection]	Have you taken therapy for active tuberculosis or latent TB infection for more than 14 days.	dropdown<br>0 No<br>1 Yes<br>2 Don't know
11	[systemic_corticosteriods]	Are you currently on any systemic corticosteriods	dropdown<br>0 No<br>1 Yes<br>2 Don't know
12	[eligible_for_enrollment]	Participant eligible for enrollment	dropdown<br>0 No<br>1 Yes
13	[eligibility_criteria_form_complete]	Section Header: *Form Status*<br>Complete?	Dropdown<br>0 Incomplete<br>1 Unveri ed<br>2 Complete

Instrument: **Baseline Risk Assessment Form** (baseline_risk_assessment_form)

14	[interview_date]	Section Header: *RESEARCHER'S INFORMATION*<br>Interview Date	text (date_dmy)
15	[study_visit_number]	Section Header: *STUDY PARTICIPANT INFORMATION*<br>Study visit number	text (number)
16	[sex]	Section Header: *SOCIO-DEMOGRAPHICS*<br>Sex	dropdown<br>1 Male<br>2 Female
17	[menstrual_period]<br>Show the eld ONLY if:<br>[sex] = '2'	Last Menstrual period	text (date_dmy)
18	[lmp_1_month]<br>Show the eld ONLY if:<br>[sex] = '2'	If last menstrual period > 1 month are you currently	dropdown<br>1 Pregnant<br>2 Menopausal<br>3 On contraceptives<br>4 Not applicable<br>5 Others
19	[specify_lmp_1_month]<br>Show the eld ONLY if:<br>[lmp_1_month] = '5'	Specify	text
20	[date_of_birth]	Date of birth	text (date_dmy), Required
21	[country_of_birth]	Country/State of birth	text
22	[educational_status]	Educational Status	dropdown<br>1 No education<br>2 Primary<br>3 Secondary/post -secondary school<br>4 Tertiary/postgraduate<br>5 Others
23	[specify_edu]<br>Show the eld ONLY if:<br>[educational_status] = '5'	Specify	text
24	[religion]	Religion	dropdown<br>1 No Religion<br>2 Christian<br>3 Islam<br>4 Others
25	[marital_status]	Marital Status	dropdown<br>1 Married<br>2 Separated<br>3 Widowed<br>4 Cohabiting<br>5 Divorced<br>6 Single
26	[employment_designation]	Section Header: *EMPLOYMENT HISTORY*<br>Employment designation	dropdown<br>1 Physicians<br>2 Nurse<br>3 Admin staff<br>4 Medical records staff<br>5 Laboratory worker<br>6 Radiology technician<br>7 Counselor<br>8 Cleaner<br>9 Dietary staff<br>10 Patient attendant<br>11 Others

27	[specify_designation]<br><br>Show the eld ONLY if:<br>[employment_designation] = '11'	Specify	text
28	[screened_for_active_tb]	Section Header: *RISK ASSESSMENT*<br><br>Have you been screened for active TB(Sputum,AFB, Gene xpert)	dropdown<br>0 No<br>1 Yes
29	[tb_control_program]	Does your facility have a TB infection control program	dropdown<br>0 No<br>1 Yes<br>2 Don't know
30	[risk_developing_active_tb]	Do you think you are at risk of developing active TB infection in your department	dropdown<br>0 No<br>1 Yes<br>2 Don't know
31	[level_of_risk]	What do you think is your level of risk (on a scale of 0-10)	text
32	[weight1]	Section Header: *PHYSICAL EXAMINATION*<br><br>Weight1(kg)	text
33	[weight2_kg]	Weight2(Kg)	text
34	[height]	Height(cm)	text
35	[blood_pressure_mm_hg]	Blood pressure(mm/hg)	text
36	[pulse_rate]	Pulse rate(per minutes)	text
37	[study_group]	Assigned Study group	dropdown<br>0 Non-Hiv group<br>1 Hiv clinic
38	[q_c_by]	Q.C. by	text
39	[baseline_risk_assessment_form_complete]	Section Header: *Form Status*<br><br>Complete?	dropdown<br>0 Incomplete<br>1 Unveri ed<br>2 Complete
Instrument: **Quantiferon TB Laboratory Form** **(quantiferon_tb_laboratory_form)**			
40	[fl_qft]	Form Login	text<br>Field Annotation: @USERNAME @READONLY
41	[frm_tim_dt_qft]	Form login date&Time	text<br>Field Annotation: @READONLY @NOW
42	[date_of_sample]	Date of Sample collection	text (date_dmy)
43	[lithium_heparin_tube]	Section Header: *SPECIMEN COLLECTION INSTRUCTION(indicate if you have done the following)FOR LABORATORY PERSONNEL ONLY*<br><br>Collect 10mls of blood into two 10mls lithium heparin tube	dropdown<br>0 No<br>1 Yes
44	[invert_10_times]	Invert 10 times firmly enough to ensure proper mixing	dropdown<br>0 No<br>1 Yes
45	[sample_tracking_form]	Complete the sample tracking form	dropdown<br>0 No<br>1 Yes
46	[lab_as_per_protocol]	Ship sample to lab as per protocol	dropdown<br>0 No<br>1 Yes

	47	[good_condition]	Section Header: *FOR LABORATORY PERSONNEL ONLY*<br>The specimen was in good condition	dropdown<br>0 No<br>1 Yes
	48	[mls_of_blood_collected]	5mls of blood for QFT was collected	dropdown<br>0 No<br>1 Yes
	49	[qft_result]	QFT Result	dropdown<br>1 Positive<br>2 Negative<br>3 Indeterminate<br>4 Not Done
	50	[quantiferon_nil]	QuantiFeron Nil(Iu/ml)	text
	51	[quantiferon_tb2]	Quantiferon TB2(Iu/ml)	text
	52	[quantiferon_tb1]	QuantiFeron TB1(Iu/ml)	text
	53	[quantiferon_mitogen]	QuantiFeron Mitogen(Iu/ml)	text
	54	[lab_scientist]	Lab scientist	text
	55	[date_of_result]	Date of result	text (date_dmy)
	56	[quantiferon_tb_laboratory_form_complete]	Section Header: *Form Status*<br>Complete?	dropdown<br>0 Incomplete<br>1 Complete

TB Infection Control Assessment FORM 6

Facility Background sheet
**Name of Health Facility**
**Level of Facility**<br>☐ National ☐ State ☐ LGA ☐ Others
**Type of facility**<br>☐ Public ☐ Private ☐ Faith based/NGO
**Level of Services at Facility**<br>☐ Teaching/Tertiary ☐ Tertiary ☐ Secondary ☐ Primary
**Time of Assessment** ☐ Baseline ☐ 12 Month
**Date: mm/dd/yyyy**

Background TB Infection Control Information

	Answer	others	Comment
1. This facility has an assigned TB infection control nurse or specialist to carry out IC activities	Yes<br>No<br>Don't Know		
2. This facility has an infection control committee	Yes<br>No<br>Don't Know		
3. An infection control assessment has occurred at this facility within the past year	Yes<br>No<br>Don't Know		
a. If yes, please state by whom			
b. Please state when this occurred			
c. Please state the outcome of the assessment			
4. This facility has a log of all employees (Physicians, nurses and others) diagnosed of TB	Yes<br>No<br>Don't Know		
5. This facility monitors employees for TB symptoms each year	Yes<br>No<br>Don't Know		
6. This facility tests employees for TB infection each year	Yes<br>No<br>Don't Know		

7. This facility provides isoniazid preventive therapy (IPT) to employees with latent TB infection	Yes<br>No<br>Don't Know		
8. Number of healthcare workers screened for TB in the past year	Yes<br>No<br>Don't Know		
a. Number of healthcare workers diagnosed with TB			
b. Number of healthcare workers completing TB treatment			

FACILITY TB RISK ASSESSMENT

Managerial	Yes	No	Don't know	Others/ Comments
1. The National infection control policy is available on site				
2. An infection control practitioner or nurse has been assigned to carry out infection control in the facility				
3. An infection control committee/team has been designated at this site.				
4. A written site-specific infection control (IC) plan has been written and is available to staff				
5. The infection control plan contains of a statement of endorsement by the facility manager				
6. A TB IC risk assessment is completed at least annually				
7. Facility design and patient flow has been assessed for the best use of space and ventilation				
8. All patients with TB disease are managed on directly observed therapy (DOTS) per the national guideline.				
9. TBIC practices are monitored daily				
10. There is a facility reporting system for all patients diagnosed with TB and referred for treatment in accordance with national policies				
11. TBIC training for all staff has been done and documented at least annually				
12. Information on TBIC is available for all patients and visitors and is offered by staff				
13. Operational research to improve TBIC measures is conducted at this site				
14. An occupational health program is in this facility				

TB Infection Control Assessment FORM 6

ADMINISTRATIVE	YES	NO	Don't know	Others/ Comments
1. Patients are routinely asked about cough when entering the facility				
2. Patients that are coughing are separated from others and are "fast tracked" to a clinician				
3. A "cough monitor" or other designated person gives cough etiquette guidance and assists with separation and triage				
4. Signage for cough etiquette is present in the clinic				
5. Supplies are available to coughing patients (tissues, cloths, masks, trash bins etc)				
6. Sputum samples are collected in a designated area and away from others				
7. Processing of sputum samples is expedited in the lab. There is a tracking mechanism to monitor turn-around time of lab. results				
8. There is a tracking mechanism to monitor turn-around time of patients within the healthcare facility				
9. Staff receive an evaluation for TB at least annually				
10. A confidential log is kept of all staff that are diagnosed with TB disease				
11. Staff are offered an HIV test annually and offered ART if they are positive				
12. HIV infected staff are re-assigned if they request				
13. INH preventive treatment is offered to HIV infected staff				
**ENVIRONMENTAL**	**YES**	**NO**	**Don't know**	**Others/ Comments**
1. Staff monitors natural and/or mechanical airflow daily (especially in waiting rooms, sputum collection room if available, and at least one exam room)				
2. Health care workers that assist during sputum collection take precautions				
3. Regular cleaning and maintenance of directional and extractor fans is conducted				
4. Servicing documentation is maintained and is available for re4view				
5. Signage is in place to keep doors and windows open when feasible				

6. If UV lighting is used, routine cleaning and maintenance is conducted and documentation log is kept.				
7. Patients waiting areas are out of doors or have good cross-ventilation				
**PERSONAL PROTECTIVE EQUIPMENT (PPE)**	**YES**	**NO**	**Don't know**	**Others/ Comments**
1. Surgical masks are available and worn by coughing patients				
2. N-95 or FFP2 respirators are readily available and used by staff				
3. Staff has been trained on proper fit of respirators and documentation of training is available				
